# Efficacy of Ivermectin, Liquid Paraffin, and Carbaryl against Mange of Farmed Rabbits in Central Kenya

**DOI:** 10.1155/2019/5092845

**Published:** 2019-11-27

**Authors:** Kennedy O. Ogolla, Joyce Chebet, Robert M. Waruiru, Peter K. Gathumbi, Paul O. Okumu, Gabriel O. Aboge

**Affiliations:** ^1^Department of Veterinary Pathology, Microbiology and Parasitology, University of Nairobi, P.O. Box 29053-00625, Kangemi, Nairobi, Kenya; ^2^Department of Public Health, Pharmacology and Toxicology, University of Nairobi, P.O. Box 29053-00625, Kangemi, Nairobi, Kenya

## Abstract

Mange is a common disease of rabbits globally, and knowledge of efficacy of drugs used in its treatment is critical for effective disease control. The current study evaluated the efficacy of three commonly used therapeutic agents in Kenya against mange. In a controlled laboratory trial, 20 adult rabbits were recruited for the study (16 of which were infested with mange, while 4 were mange-free). The 16 mange-infested rabbits were randomly allocated into 4 treatment groups each consisting of 4 rabbits, while 4 mange-free rabbits formed the negative control group. Treatments were administered as follows: group 1 (G1) received two ivermectin injections at an interval of 14 days, group 2 (G2) was treated with a combination of carbaryl and liquid paraffin applied every other day up to the end of the experiment, group 3 (G3) was treated with liquid paraffin droplets applied daily until the lesion cleared, while group 4 (G4, infected-untreated) received distilled water applied topically on their ears and group 5 (G5, uninfected-untreated negative control) was not treated with any preparation. The lesions were scored and sampled daily to check the viability of the mites. A field efficacy trial of the test compounds was performed using 105 mange-infested rabbits. The results revealed that all the test agents: ivermectin, liquid paraffin, carbaryl-water, and carbaryl-liquid paraffin combination were effective against mange, recording the lesion score of zero for psoroptic mange by day 21 in the laboratory and field trials. Lesion scores in the treated groups were significantly reduced (*p* < 0.05) at the termination of study compared with those of the positive control group in the laboratory trial. A point-biserial correlation revealed a strong association (*r*_pb_ = 0.79, *p* < 0.05) between the presence of viable mites and degree of psoroptic lesions in the field trial.

## 1. Introduction

Skin conditions are among the most common diseases affecting pet, commercial, and laboratory rabbits in Kenya [1, 2]. The common causes of these diseases are ectoparasites such as fleas, lice, and mites [2, 3]. Mites cause mange, a highly contagious disease affecting several species of animals [4]. Mange is spread easily by direct contact between sick and healthy rabbits or indirectly through fomites [5]. *Psoroptes cuniculi*, *Sarcoptes scabiei* var. *cuniculi*, and *Cheyletiella parasitovorax* are the commonly reported mites infesting rabbits in Kenya [2]. *Psoroptes cuniculi* is a large surface mite that causes psoroptic mange. Affected rabbits primarily present with clinical signs of intense pruritus, head shaking, erythema, and crusty, scaly, and scabby lesions on the inner side of the pinnae and on the external ear canal [6]. In extreme cases, rabbits may present with drooping ears, foul-smelling discharges emanating from the external ear canal, and occasionally pain on palpation of the ear which is generally limited to the ear but can also be diffuse [5, 7]. On the other hand, *Sarcoptes scabiei* var *cuniculi* is a burrowing mite that causes sarcoptic mange characterized by extreme scratching, alopecia, crusts, scales, and erythema on the skin of the margins of pinna, lips, nose, legs, and areas adjoining the genitalia [8, 9]. In chronic cases, rabbits may present with anorexia, lethargy, and loss of body condition, all of which sporadically results in death [8]. *Cheyletiella parasitovorax* is a large, fast-moving, non-burrowing mite, which can be seen moving on the skin [5]. Infested rabbits show signs of pruritus, scratching, crusting, scales on the skin, and alopecia, among others [5]. *Sarcoptes scabiei* var. *cuniculi* and *Cheyletiella parasitovorax* have zoonotic potential [10].

Mange in rabbits has been treated effectively using chemotherapeutic and non-chemotherapeutic methods [11]. Common chemotherapeutic agents used include avermectins such as abamectin, doramectin, ivermectin, and selamectin [11–13], topical permethrin [14], carbamates, benzyl benzoate, and sulphur-based compounds [15]. Ivermectin is a macrolide that acts by opening the invertebrate-specific glutamate-gated chloride ion channels in the membrane controlled by GABA, which normally blocks the transmission of signals from the central command interneurons to the peripheral motor neurons, thereby reducing the muscle membrane resistance [16]. On the contrary, carbaryl is a broad-spectrum insecticide in the carbamate group [17]. Carbaryl inhibits the action of acetyl cholinesterase enzyme, which hydrolyses acetylcholine into choline and acetic acid, thus disrupting smooth transmission of nerve impulses in target ectoparasites [18]. Botanicals such as garlic extract [19, 20] and essential oils from plants [21, 22] and other nonchemical remedies like paraffin oil [4] have also been used with varied efficacy against mites. Studies have postulated that oils act by making direct contact with the mites and also by blocking the opening to tunnels within the stratum corneum through which the buried mites breathe, thereby suffocating the parasites [4, 23]. In Kenya, there are no rabbit-specific drugs for the treatment of mange, and the ones available are extrapolated from other species particularly dogs (ivermectin) and poultry (carbaryl). These drugs are used with little knowledge of their efficacy, safety, or appropriate dosages in farmed rabbits. While resistance to ivermectin against some *Sarcoptes scabiei* species [24] and side effects [25] have been reported elsewhere, there are no documented evidence of its resistance in Kenya. The present study is the first to evaluate the comparative efficacy of ivermectin, carbaryl, and liquid paraffin against mange in farmed rabbits in Kenya. Since the efficacy of ivermectin in treatment of mange of rabbits globally is well proven [9], it is prudent to benchmark other treatments of the disease with it as was the case in this study. The three antimange agents were chosen because they are the most commonly used by rabbit farmers in Central Kenya as reported in a recent study [3].

## 2. Materials and Methods

### 2.1. Description of the Study

Randomized controlled laboratory and field trials were undertaken to determine the comparative efficacy of ivermectin, carbaryl, and liquid paraffin against mange of rabbits. In the laboratory trial, twenty adult rabbits of mixed breeds obtained from commercial rabbit farms which had never been treated with any antiparasitic drugs were used. Sixteen rabbits were naturally infested with psoroptic mange, while four were mange-free. The mange-infested rabbits were randomly allocated to 4 treatment groups: group 1 (G1), group 2 (G2), group 3 (G3), and group 4 (G4), each consisting of 4 rabbits. Group 5 (G5) composed of mange-free farmed rabbits served as the uninfected-untreated negative control group. In the field trial, 95 (91.3%) psoroptic-infested rabbits and 9 (8.7%) sarcoptic-infested rabbits were used. They were randomly allocated into 3 groups (treatment group 1 (TG1), treatment group 2 (TG2), and treatment group 3 (TG3)) each with 35 rabbits. All experimental rabbits were housed individually in widely separated cages to avoid direct contact in a house with adequate natural ventilation and lighting.

### 2.2. Clinical Examination and Scoring of Skin Lesions

The rabbits were examined for clinical signs and pathological lesions consistent with mange before and after commencement of treatment. The presence of live mites was confirmed by microscopic examination, and lesions examined included alopecia, ear scabs, erythema, crusts, dandruff, and exudation. Further examination after commencement of treatment was done at 3-day interval until day 21. Thereafter, the last examination was done 7 days after the last treatment (day 28). Rabbits with psoroptic mange were scored for severity of infestation using criteria described by Pan et al. [26], where 0 = ear appears normal/no evident lesion, 1 = lesions confined to the ear canal, 2 = lesions confined to lower third of the auricle, 3 = lesions confined to lower 2/3rd of the auricle, and 4 = extensive lesions covering an area greater than 2/3rd of the auricle.

### 2.3. Parasitological Examination

Before recruitment into the study, deep and superficial skin scrapings and ear scab samples were collected from the rabbits to confirm sarcoptic and psoroptic mite infestation, respectively. Samples were then collected on day 0 (start of the treatment day) and afterwards on days 7, 14, and 21 during the treatment period, and last sampling was done on day 28 (one week after treatment termination) to assess response to treatment. During the field trial, additional sampling was done on day 56 to check for any relapse. Samples were placed in labelled sterlin tubes until the time of analysis. Each sample was divided into two portions where one was digested with 10% potassium hydroxide and examined under a light microscope (Germany Leica DM750 biomicroscope) for identification of the mites. The other portion was soaked in warm physiological saline (32°C), debris was filtered out, and then examined under a microscope for presence or absence of live mites as described by OIE [27]. Viability of mites was based on slight modification of criteria described by Pap et al. [28], where mites with total mobility or active movement of two or more limbs were recorded as alive, while those with active movement in less than two limbs were recorded as dead. Mites were categorized into their respective genera based on their morphological characteristics. The rabbits were also fur-combed to check for other ectoparasites such as fleas, lice, and flea dirt. This study was approved by the Biosafety, Animal Use and Ethics Committee of the University of Nairobi (reference no. FVM BAUEC/2018/144).

### 2.4. Laboratory Evaluation of the Acaricides Efficacy

The rabbits in G1 were subcutaneously injected with ivermectin (Supermec®, Bimeda, Kenya) at 400 *μ*g/kg body weight on days 0 and 14 as recommended by the manufacturer for dogs. On day 0, G2 rabbits were treated topically with 5–8 drops (0.5 ml) of liquid paraffin (Liquid Paraffin®, Farmvet Africa Ltd, Kenya) to wet the lesions. Thereafter, the wetted lesions were dusted with carbaryl (Dudu Dust®, Bayer, Germany) to cover the entire lesion. This treatment was repeated at 1-day interval until day 21. For G3 rabbits, 9 drops (0.6 ml) of liquid paraffin was applied topically on the lesions on a daily basis until day 21. The G4-infested rabbits were treated topically with 8 drops (0.5 ml) of distilled water applied daily until day 21 and therefore served as the positive control. The G5 non-infested healthy rabbits were not treated with any preparation and served as the negative control. All the rabbits in G4 (non-treated infested rabbits) received full dose of ivermectin injection at termination of the study in line with animal welfare requirements. Efficacy of the test compounds was based on elimination of sarcoptic mange lesions, reduction of lesion scores in psoroptic mange, and absence of viable mites on microscopic examination.

### 2.5. Field Evaluation of the Acaricides Efficacy

Rabbits in TG1 were treated with subcutaneous injections of ivermectin at 400 *μ*g/kg body weight on day 0 and repeat injection administered after 14 days; rabbits in TG2 were dusted with carbaryl to cover the lesion after applying 5 drops of water to wet the lesion on day 0 and then after every 3 days for a period of 21 days; lastly, 9 drops of liquid paraffin were applied on the lesions on day 0 and then after every three days up to day 21 in TG3. Efficacy of test compounds was determined as described for the laboratory trial.

### 2.6. Data Management

Psoroptic lesions of the ears were scored in the laboratory and field trials. In the field trial, though each treatment group had 35 rabbits, only 29 rabbits with psoroptic mange lesions were scored. Data were analyzed using SPSS programme. Descriptive statistic results were presented as mean ± SEM, and significance levels itemized at *p* < 0.05. Kruskal–Wallis one-way analysis of variance in SPSS was conducted to examine the difference in lesion scores depending on the test agents administered. Dunn–Bonferroni test for pairwise comparisons was conducted post hoc to illustrate the significant differences between treatment groups. A point-biserial correlation was then run to check the relationship between the presence of viable mites and the extent of psoroptic mange lesion scores.

## 3. Results and Discussion

### 3.1. Parasitological Examination

**Figure 1:**
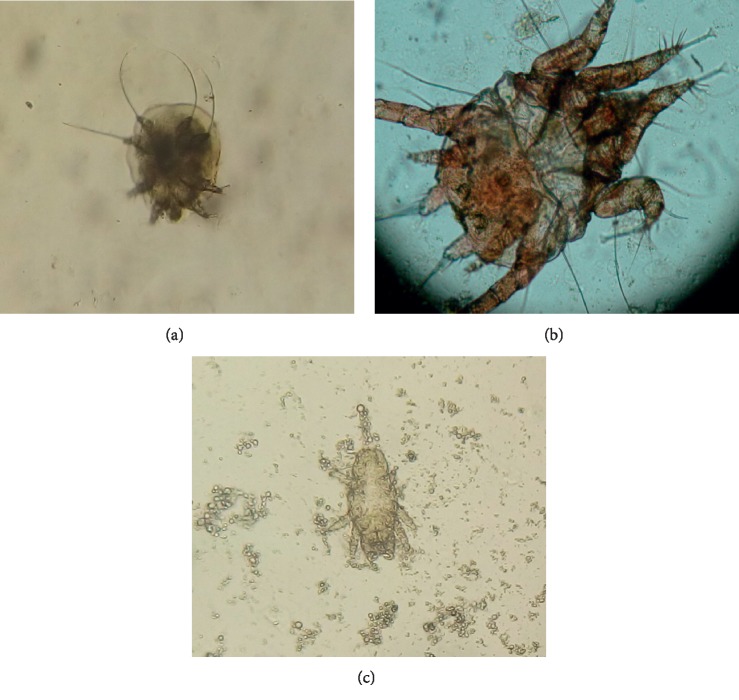
Parasites recovered during the laboratory and field trial of antimange agents: (a) *Sarcoptes scabiei* var. *cuniculi* at ×100, (b) a male *Psoroptes cuniculi* at ×100, and (c) *Cheyletiella parasitovorax* at ×100.

Only *Psoroptes cuniculi* was recovered from the rabbits in the controlled laboratory trial, while *P. cuniculi* (a), *Sarcoptes scabiei* var *cuniculi* (b), and *Cheyletiella parasitovorax* (c) were recovered from the rabbits during the field trial. The mites recovered during the two trials are shown in Figure 1. High prevalence of psoroptic and sarcoptic mange in rabbits has been reported in other studies [28–30]. The rabbit that had mixed infestation manifested signs of scratching, dandruff, and alopecia on the back. McTier et al. [12] and Kurtdede et al. [13] have described similar clinical signs during infestation of rabbits by mites. There was a strong correlation (*r*_pb_ = 0.79, *p* < 0.05) between the presence of viable mites and the extent of psoroptic mange lesions assessed through lesion scores.

### 3.2. Antimange Efficacy Trials

**Figure 2:**
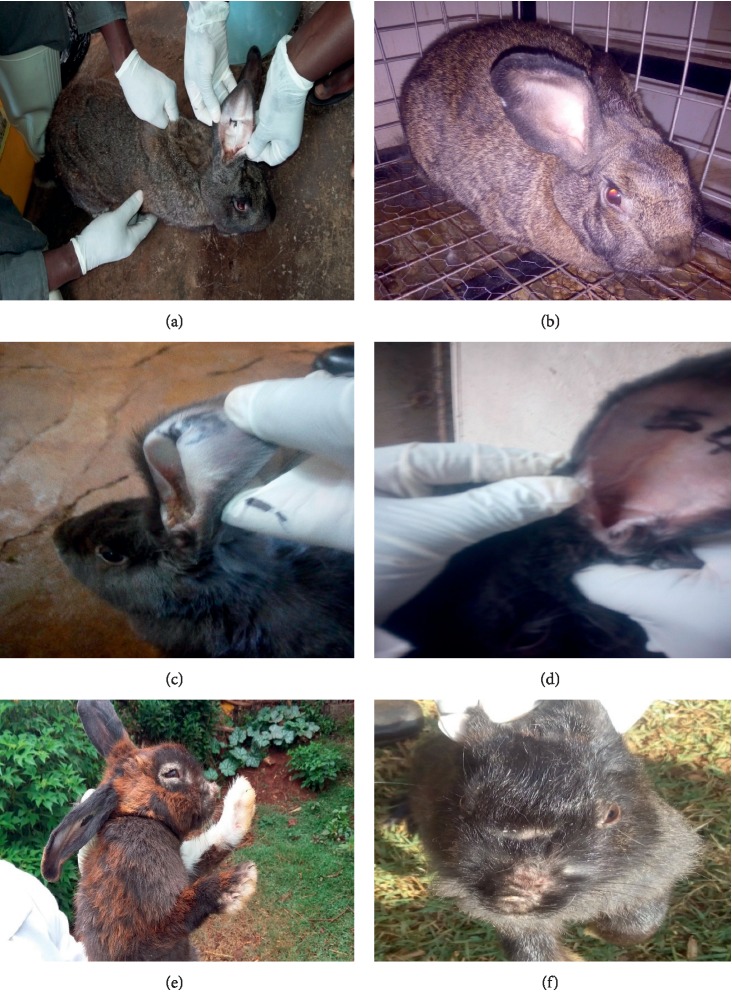
A rabbit with psoroptic mange (a) and the same rabbit after successful treatment with ivermectin (b). A rabbit with ear canker before (c) and after (d) treatment with carbaryl. A rabbit with sarcoptic mange around the mouth and nose (e) before treatment and (f) after treatment with liquid paraffin.

Ivermectin, liquid paraffin, carbaryl-liquid paraffin combination as used in the laboratory trial, and carbaryl-water combination used during the field study were all efficacious against psoroptic and sarcoptic mange. Efficacy was manifested by reduction of lesions in the course of treatment as presented in Tables 1 and 2 and lack of viable mites at study termination (Tables 3 and 4). There was a significant difference (*p* = 0.001) in psoroptic mange lesion scores 7 days (day 28) after termination of treatment between the positive control (infected not treated) group and the treated groups in the controlled laboratory trial. All the treated rabbits showed progressive recovery manifested by improved skin texture, healing of skin lesions, resolution of erythema, and fading of crusts. By day 21, all rabbits treated with liquid paraffin and carbaryl-liquid paraffin combination were negative for mites, while only one rabbit from the ivermectin treatment group had partially resolved clinical lesions (Figure 2(a)), but the mites recovered were non-viable (dead) in the laboratory trial as presented in Figure 2 and Table 3. Mite infestation was eliminated 7 days after termination of treatment (day 28) in all the treated rabbits in the laboratory trial. In contrast, the infested-untreated (positive control) rabbits presented with worsening lesions during the entire study duration. All the rabbits in the control group remained positive up to termination of the experiment. In the present study, use of liquid paraffin alone cleared psoroptic mange by day 21 in the laboratory trial, while only dead sarcoptic and psoroptic mites were recovered 7 days after the last treatment in the field trial. A relapse of infestation was, however, observed 37 days after termination of treatment with liquid paraffin in 7 out of 34 rabbits treated for psoroptic mange and 1 rabbit treated for sarcoptic mange, all of which had viable mites on day 56 (Table 4). The relapse may be attributed to possible reinfestation and the fact that the action of liquid paraffin is contact dependent with no residual effects in the body once application is stopped. Liquid paraffin like other oils has been reported to act by making direct contact with the mites and also blocking the opening to tunnels within the stratum corneum through which the buried mites breath, thereby suffocating the parasites [4, 23]. Efficacy of non-conventional therapies against mange has likewise been demonstrated for plant essential oils [31, 32] and for other plant extracts [33]. In the laboratory trial, carbaryl-liquid paraffin combination recorded the fastest action in clearing mite infestations and associated lesions compared with the use of carbaryl-water combination (as used in field trial), liquid paraffin, and ivermectin. The effectiveness of carbaryl against mites in our study agrees with findings reported by Ebrahimi et al. [34]. However, the labour-intensive nature of this treatment regimen that requires initial wetting of the lesion before carbaryl can be applied coupled by the extra cost of carbaryl may limit its adoption compared with the cheaper use of liquid paraffin alone. Further, carbaryl poses ecological and toxicological concerns that limit its application in control of ectoparasitism. The use of liquid paraffin against rabbit mange should be promoted as it is easily accessible, relatively affordable, has no known side effects, and will contribute to reduction in use of antiparasiticides such as ivermectin and carbaryl, thereby minimising acaricide resistance and accumulation of their residues in animal products.

Similarly, findings of the present study confirmed efficacy of two injections of ivermectin administered at 400 *μ*g/kg after 14 day interval against local psoroptic and sarcoptic mite isolates. There was no relapse of mite infestation in groups of rabbits treated with ivermectin or carbaryl-water combination, during the field trial. Effectiveness of avermectins in controlling rabbit mange has been reported in previous studies for moxidectin [35], selamectin [12, 13, 36], doramectin [37], and ivermectin [14, 38]. Since ivermectin has proven efficacy against other ectoparasites and some endoparasites such as helminths, rabbit farmers targeting control of both ectoparasites and endoparasites prefer it to other drugs. However, with the development of resistance already reported in other regions, ivermectin should be used judiciously in treatment of rabbit mange. Liquid paraffin provides an interim alternative to break continued use of ivermectin, hence minimising the risk of drug resistance. No adverse reaction was observed with any of the treatment regimens in this study.

## 4. Conclusion

Based on the dosages and durations of treatment used in this study, ivermectin, liquid paraffin, and carbaryl were effective against sarcoptic and psoroptic mite-infested rabbits. The authors recommend adoption of liquid paraffin as a farmer's level interlude remedy for rabbit sarcoptic and psoroptic mange. Liquid paraffin has a wide safety margin and does not carry the risk of drug resistance. Furthermore, it is readily available, affordable, and easily administered at a farm level. Liquid paraffin also offers a break in the use of conventional synthetic treatments such as ivermectin which minimizes the development of drug resistance.

## Figures and Tables

**Table 1:** Mean lesion scores in the controlled laboratory efficacy trial of antimange agents in Central Kenya.

Group	Lesion scores after treatment					
	Day 1	Day 7	Day 14	Day 21	Day 25	Day 28
Ivermectin	3.25 ± 0.48^b^	0.75 ± 0.25^ab^	0.5 ± 0.29^ab^	0.25 ± 0.25^ab^	0.25 ± 0.25^a^	0.0 ± 0.0^a^
Liquid paraffin	2.0 ± 0.71^ab^	0.75 ± 0.25^ab^	0.25 ± 0.25^a^	0.25 ± 0.25^ab^	0.0 ± 0.0^a^	0.0 ± 0.0^a^
Negative control	0.0 ± 0.0^a^	0.0 ± 0.0^a^	0.0 ± 0.0^a^	0.0 ± 0.0^a^	0.0 ± 0.0^a^	0.0 ± 0.0^a^
Positive control	1.75 ± 0.48^ab^	1.75 ± 0.48^b^	1.75 ± 0.48^b^	1.5 ± 0.5^b^	1.75 ± 0.48^b^	1.75 ± 0.48^b^
Carbaryl	2.0 ± 0.0^ab^	0.5 ± 0.29^ab^	0.25 ± 0.25^a^	0.0 ± 0.0^a^	0.0 ± 0.0^a^	0.0 ± 0.0^a^
*X* ^2^(4)	12.66	10.60	10.75	12.68	15.65	18.737
*p* value	0.013	0.031	0.029	0.013	0.004	0.001

Four rabbits per treatment group were scored for psoroptic mange lesions using criteria described by Pan et al. [26]. Kruskal–Wallis one-way analysis of variance in SPSS was conducted to examine the difference in lesion scores depending on test compounds administered. Dunn–Bonferroni test for pairwise comparisons was conducted post hoc to illustrate the significant differences between treatment groups. Values within a column without a common superscript are significantly different at *p* < 0.05.

**Table 2:** Mean lesion scores in the field efficacy trial of antimange agents in Central Kenya.

Group	Lesion scores during treatment						
	Day 0	Day 3	Day 9	Day 15	Day 21	Day 28	Day 56
Ivermectin	2.17 ± 0.15	1.57 ± 0.13	1.09 ± 0.12	0.71 ± 0.11	0.50 ± 0.11	0.24 ± 0.09	0.13 ± 0.05
Liquid paraffin	1.89 ± 0.12	1.09 ± 0.10	0.54 ± 0.10	0.27 ± 0.10	0.11 ± 0.06	0.03 ± 0.03	0.20 ± 0.12
Carbaryl	2.0 ± 0.13	1.286 ± 0.097	0.74 ± 0.10	0.50 ± 0.10	0.31 ± 0.09	0.23 ± 0.083	1.17 ± 0.06
*X* ^2^(2)	0.69	0.328	3.153	2.189	2.608	0.680	0.913
*p* value	0.709	0.849	0.207	0.335	0.271	0.712	0.634

A total of 29 rabbits per treatment group were scored for psoroptic mange lesions using criteria described by Pan et al. [26]. Kruskal–Wallis one-way analysis of variance in SPSS was conducted to examine the difference in lesion scores depending on test compounds administered.

**Table 3:** Viability of mites in the laboratory efficacy trial of ivermectin, liquid paraffin, and carbaryl-liquid paraffin combination against rabbit psoroptic mange.

Treatment group	Number of rabbits with and without *Psoroptes cuniculi* during the study period									
	Day 0 (treatment)		Day 7		Day 14		Day 21		Day 28	
	+ve	−ve	+ve	−ve	+ve	−ve	+ve	−ve	+ve	−ve
Ivermectin (G1)	^v^(4)	0	^v^(2) ^d^(1)	1	^d^(3)	1	^d^(3)	1	^d^(0)	4
Liquid paraffin (G2)	^v^(4)	0	^v^(4)	0	^d^(2)	2	^d^(0)	4	^v^(0)	4
Carbaryl-liquid paraffin (G3)	^v^(4)	0	^v^(1) ^d^(1)	2	^d^(1)	3	^d^(0)	4	^d^(0)	4
Positive control (G4)	^v^(4)	0	^v^(4)	0	^v^(4)	0	^v^(4)	0	^v^(4)	0
Negative control (G5)	^v^(0)	4	^v^(0)	4	^v^(0)	4	^v^(0)	0	^v^(0)	0

Note: +ve = rabbits with mites; −ve = rabbits without any mite on microscopic examination of samples. Items in parentheses represent number of rabbits. Numbers with the superscript “v” are rabbits with viable (live) mites, while those with the superscript “d” are rabbits with nonviable (dead) mites.

**Table 4:** Viability of mites in the field efficacy trial of ivermectin, liquid paraffin, and carbaryl in rabbits naturally infested with mange in Central Kenya.

Treatment group	Number of rabbits with and without *Psoroptes*, *Sarcoptes*, and *Cheyletiella* mites before, during, and after treatment									
	Day 0		Day 7		Day 14		Day 28		Day 56	
	+ve	−ve	+ve	−ve	+ve	−ve	+ve	−ve	+ve	−ve
Ivermectin (TG1)	^v^(29)Pc	0	^v^(21)Pc	8	^d^(15)Pc	14	^d^(3)Pc	26	^d^(1)Pc	27
	^v^(5)Ss	0	^v^(4)Ss	1	^d^(1)Ss	4	^d^(0)Ss	5	(0)Ss	5
	^v^(1)Cp	0	^v^(1)Cp	0	(0)Cp	1	(0)Cp	1	(0)Cp	1
Carbaryl-water (TG2)	^v^(32)Pc	0	^v^(21)Pc	11	^d^(7)Pc	25	^d^(5)Pc	27	^d^(1)Pc	31
	^v^(3)Ss	0	^v^(1)Ss	2	(0)Ss	3	(0)Ss	3	(0)Ss	3
Liquid paraffin (TG3)	^v^(34)Pc	0	^v^(22)Pc	12	^d^(13)Pc	21	^d^(3)Pc	31	^v^(7)Pc	27
	^v^(1)Ss	0	^v^(1)Ss	0	^d^(1)Ss	0	^d^(1)Ss	0	^v^(1)Ss	0

Note: +ve = rabbits with mites; −ve = rabbits without any mite on microscopic examination of samples. Items in parentheses represent number of rabbits. Pc, *Psoroptes cuniculi*; Ss, *Sarcoptes scabiei* var *cuniculi*; Cp, *Cheyletiella parasitovorax*. Numbers with the superscript “v” are rabbits with viable (live) mites, while those with the superscript “d^”^ are rabbits with nonviable (dead) mites.

## Data Availability

Data on this experiment can be accessed at https://data.mendeley.com/datasets/fs9vsw6h4c/1.
